# Biomarker Detection in Early Diagnosis of Cancer: Recent Achievements in Point-of-Care Devices Based on Paper Microfluidics

**DOI:** 10.3390/bios13030387

**Published:** 2023-03-15

**Authors:** Bilge Asci Erkocyigit, Ozge Ozufuklar, Aysenur Yardim, Emine Guler Celik, Suna Timur

**Affiliations:** 1 Department of Biotechnology, Institute of Natural Sciences, Ege University, Izmir 35100, Turkey; 2 Department of Bioengineering, Institute of Natural Sciences, Ege University, Izmir 35100, Turkey; 3 Department of Bioengineering, Faculty of Engineering, Ege University, Izmir 35100, Turkey; 4 Department of Biochemistry, Faculty of Science, Ege University, Izmir 35100, Turkey; 5 Central Research Test and Analysis Laboratory Application, Research Center, Ege University, Izmir 35100, Turkey

**Keywords:** paper-based microfluidics, biomarker detection, cancer biomarkers, in vitro diagnostics, point-of-care testing (POCT)

## Abstract

Microfluidics is very crucial in lab-on-a-chip systems for carrying out operations in a large-scale laboratory environment on a single chip. Microfluidic systems are miniaturized devices in which the fluid behavior and control can be manipulated on a small platform, with surface forces on the platform being greater than volumetric forces depending on the test method used. In recent years, paper-based microfluidic analytical devices (μPADs) have been developed to be used in point-of-care (POC) technologies. μPADs have numerous advantages, including ease of use, low cost, capillary action liquid transfer without the need for power, the ability to store reagents in active form in the fiber network, and the capability to perform multiple tests using various measurement techniques. These benefits are critical in the advancement of paper-based microfluidics in the fields of disease diagnosis, drug application, and environment and food safety. Cancer is one of the most critical diseases for early detection all around the world. Detecting cancer-specific biomarkers provides significant data for both early diagnosis and controlling the disease progression. μPADs for cancer biomarker detection hold great promise for improving cure rates, quality of life, and minimizing treatment costs. Although various types of bioanalytical platforms are available for the detection of cancer biomarkers, there are limited studies and critical reviews on paper-based microfluidic platforms in the literature. Hence, this article aims to draw attention to these gaps in the literature as well as the features that future platforms should have.

## 1. Introduction

Microfluidic systems are one of the fastest growing areas of research today. Due to their miniaturization, they are frequently used in different fields of study ranging from food safety, diagnostics, and environmental studies to organ mimetics. They also contribute to interdisciplinary communication and information exchange as they combines different techniques and technologies in a single platform [1]. Microfluidic systems gain more powerful and beneficial features when combined with paper-based systems. In addition to being portable and economical to perform analyses with a small sample volume, microfluidics has the capacity to store biological materials such as antibodies and aptamers between cellulose fibers for a long time by keeping them stable [2,3]. The capillary action of the sample without the need for an instrument pump enables multiple tests [4].

Cancer, which is frequently encountered in our age, is analyzed by imaging techniques and morphological investigation of suspicious cells taken from the patient. Since these methods have low sensitivity, they do not have sufficient capacity to distinguish between normal and abnormal cells. Paper-based microfluidic analytical devices (μPADs) are classified as promising diagnostic tools to detect cancer through biomarkers [5]. Early diagnosis of cancer by developing these test platforms accessible to everyone will make a great contribution to preventing the progression of the disease.

The fabrication of paper-based biosensors is relatively simple and can be accomplished with standard laboratory equipment. The use of paper as a substrate provides several advantages including low cost, disposability, and biocompatibility. Furthermore, paper is readily available and can be easily modified to incorporate various chemical or biological agents. The concept of paper-based biosensors is not new. For instance, lateral flow assays (LFA) are a type of paper-based diagnostic test that is widely used for the detection of various analytes, including proteins, nucleic acids, and small molecules. These assays are simple to use, rapid, and inexpensive, making them suitable for point-of-care (POC) testing in a variety of settings. The principle of LFAs is based on the movement of a liquid sample along a paper strip that contains various immobilized reagents. The sample interacts with these reagents, resulting in the formation of a visible signal that indicates the presence or absence of the target analyte [6]. The detection mechanism in paper-based biosensors can be based on various principles, including colorimetry, electrochemistry, and fluorescence. In a typical colorimetric biosensor, a paper strip is coated with a receptor that specifically binds to the target molecule of interest. The binding event induces a visible color change that can be detected with the naked eye or a simple device such as a smartphone camera. Recent advances in microfluidics have enabled the integration of complex fluidic systems into paper substrates, allowing for the analysis of biological samples in a portable and inexpensive format. On the other hand, microfluidics enables precise control of fluid movement, allowing for the manipulation of small volumes of liquid, and the creation of complex assays. In addition, multiple channels, which can be created on the paper base, allow the simultaneous determination of different analytes [2]. Signals such as electrochemical, optical, and chemiluminescence can be generated with various probes and integrated with different detection and readout techniques. As a result, μPADs may gain a new dimension in the field of biosensors by incorporating paper and smart devices into microfluidic systems.

The integration of µPADs in POC settings has the potential to revolutionize cancer diagnosis and management, particularly in resource-limited settings where traditional diagnostic tools may not be available. The ability to rapidly and accurately detect cancer biomarkers using a low-cost and portable device can provide access to early detection and monitoring of cancer in remote or underserved areas. However, challenges such as sensitivity, specificity, and reproducibility need to be addressed before this technology can be widely adopted in clinical practice.

Microfluidic biosensors and cancer biomarkers have been the focus of many recent papers. There are reviews centered around many topics such as electrochemistry, electrochemiluminescence, commercialization, manufacturing techniques, and microfluidic chips [7,8,9,10]. As a departure from the overview of paper-based biosensors provided by existing articles, we have reviewed studies in the field of microfluidic paper-based biosensors and discussed their contribution to public health by addressing their relevance to POC, point-of-need (PON), and telemedicine.

**Figure 1:**
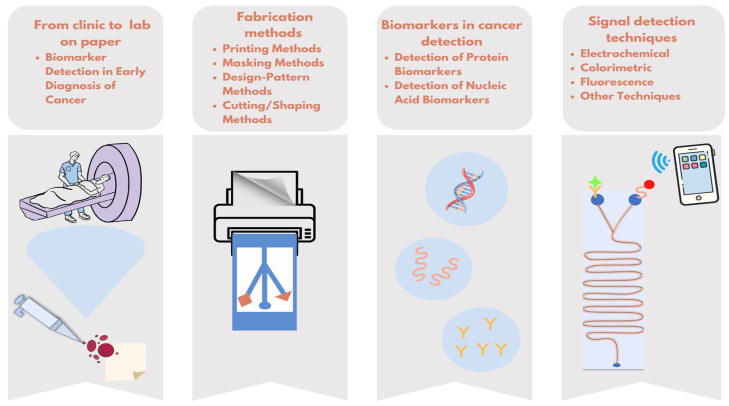
Overview of simplifying cancer diagnostics with paper-based microfluidic tests.

In this review, μPADs, which are promising for patients and healthcare professionals, will be examined in terms of target biomarkers, production techniques, and measurement methods (Figure 1).

## 2. Biomarker Detection in Early Diagnosis of Cancer

Biomarkers are generally defined as characteristics that can be measured scientifically to establish if a biological condition in an organism is normal or abnormal [11]. As a result of evolving needs and technological advancements over the past ten years, the World Health Organization has broadened this definition to include “any content, formation, or process that can be monitored in the body or its products and that influences or predicts the outcome or disease incidence”. Biomarkers include DNA, RNA, proteins, peptides, chemical compounds, etc. [11,12,13,14]. They are also associated with specific molecular pathway deregulations and/or cancer pathogenesis in the process of supporting the use of therapeutic/interventional strategies. Cancer biomarkers can be classified into three categories based on their application. The first of them are predictive biomarkers, which provide responses to a specific therapeutic process. The second type of biomarker is prognostic biomarkers, which reveal cancer risks (cancer recurrence or disease progression in the future) based on clinical results. The marker in the final classification is a diagnostic biomarker that is used to evaluate whether a patient has a particular cancer situation [11].

Imaging and histopathological analysis are two common methods for detecting and monitoring cancer. The standard procedure varies depending on the type of cancer and involves performing multiple imaging protocols prior to confirming the type and staging of cancer through tissue biopsies. Liquid biopsies have gained popularity among researchers to overcome these limitations. Exosomes, circulating tumor DNAs (ctDNAs), and circulating tumor cells (CTCs) are just a few of the many biomarkers found in a liquid biopsy that can be used to track cancer progression. Furthermore, it is simple to collect multiple liquid biopsies at regular intervals to track the progression of cancer and metastatic disease in patients in real time. Before the characterization of the biomarkers via analytical methods such as enzyme-linked immunosorbent assay (ELISA), immunohistochemistry (IHC), and gene sequencing, they need to be isolated from the liquid biopsies. However, these methods, which require skilled labor, are costly, time-consuming, and do not support real-time analysis. Biosensors have risen to prominence as a solution to these issues [15,16]. These biomarkers are currently diagnosed using various techniques such as electrochemical, chemiluminescent, colorimetric, and fluorescent methods in the biosensing area [13,17,18]. Imaging, genetic, epigenetic, proteomic, and glycomic analyses of blood or serum samples have all been used to aid in cancer diagnosis, prognosis as well as epidemiology [11,19].

There are several commercial kits available to support cancer diagnosis. These no longer solely serological kits (Accu-Tell CEA Rapid Test Cassette, rapid prostate cancer test by Zhejiang Orient Gene, etc.) have evolved into direct PCR-based tools (lung cancer rapid diagnostic test by Lepu Medical Technology, cancer rapid diagnostic test by SPACEGEN, etc.) and nucleic acid-based LFAs (NABLFAs), which combine molecular detection and the principles of immunochemical visualization [20,21]. The same principles as those behind LFAs are used to develop NABLFAs, but rather than using LFAs as test strips, the analysis of target molecules is based on hapten binding and amplification. In comparison to conventional PCR techniques, developing NABLFAs for use in basic research and clinical applications has become quite attractive because they take less time to complete and provide a simpler analysis. Moreover, they can perform colorimetric detection of PCR products without the use of any additional tools or trained personnel [20]. When static phase-based LFAs are scrutinized, incorporating these systems into microfluidic systems will result in more accurate measurements with smaller volumes due to some constraints, such as the use of additional chemicals, false positives, or storage stability. Thus, the use of additional chemicals and commercial production costs for the application can be decreased with the advancement of μPADs, while the applicability and accessibility in territories with limited resources can be improved.

## 3. Design and Working Principles of μPADs

As a substrate material, paper has unique properties such as liquid transfer based on capillary effect without any external force, ease of design, and a high surface-area-to-volume ratio that improves detection limits. In addition to these features, it has become the most widely used material in point-of-care (POC) devices due to its cost-effectiveness, ease of production, and operation. The type of paper, which has a significant effect on performance and fluidic transport, is chosen according to the users’ application. 

Paper is made from cellulose or nitrocellulose, which are hydrophilic in nature. The design of μPADs starts with the creation of a hydrophobic barrier on a paper surface via different fabrication methods. Initially, a system consisting of hydrophilic paper and channels is created. Thus, the sample moves through the channels with the help of capillary action without the need for any external force. Nevertheless, for many applications, the velocity distribution in a microfluidic network is indeed an important element to control [22].

An important parameter to control system design and function in microfluidics is the understanding of fluid flow behavior in microchannels [2]. The fluids utilized in those structures are typically liquids. The basic principle of microfluidic paper-based systems is to create hydrophilic–hydrophobic barriers on chromatography or filter paper to design fluid channel networks. The fluid follows these channels through capillary forces, the so-called Lucas–Washburn and Darcy equations [23,24]. Controlled liquid flow is ensured by trapping the liquids in the created microchannels. Liquids can be delivered through channels using a variety of microfluidic patterns with diverse designs, such as two-dimensional (2D) and three-dimensional (3D).

### 3.1. Fabrication Methods of μPADs

The techniques used to fabricate paper microfluidic devices rely on patterns of hydrophobic areas. These features are necessary in order to enable sophisticated analytical functions on paper. Microfluidic technology differs from traditional paper-based tests in several properties. Microfluidics is more accurate and can be applied more quickly than traditional tests. Fabrication methods can be examined under three main categories of printing, masking, and design-pattern methods. These different types of fabrication techniques each have their own special benefits. For instance, wax printing-based fabrication is a 2D technique, which is a quick and easy process that can be performed in 5–10 min, with the required design of microchannels. On the other hand, the drawbacks include the requirement of expensive equipment and the necessity for further heating following the wax deposition procedure [2,23,25]. Although wax-printed devices were easy to manufacture, there was a desire to develop more complex devices with advanced functionality. For this purpose, the most commonly used production methods for creating microfluidic channels are based on creating a barrier on the paper using different devices or selectively cutting/removing the paper. Moreover, μPADs can be produced using many methods such as plasma treatment, inkjet printing [26,27], paper cutting, wax printing [28,29], photolithography, etc. Different μPAD manufacturing methods for cancer biomarker detection are given in Table 1. 

### 3.2. Two-Dimensional (2D) Microfluidics

Most μPADs are fabricated using the 2D methods. Based on the idea of sequentially delivering one or more reagents to a region under capillary flow control, 2D μPADs may be used to guide fluid flow efficiently and cost-effectively via lateral flow. However, in biological testing, the sequential addition of reagents is time-consuming. This process changes certain areas (or lines) of cellulose paper from hydrophilic to hydrophobic.

#### 3.2.1. Printing Methods

**Figure 2:**
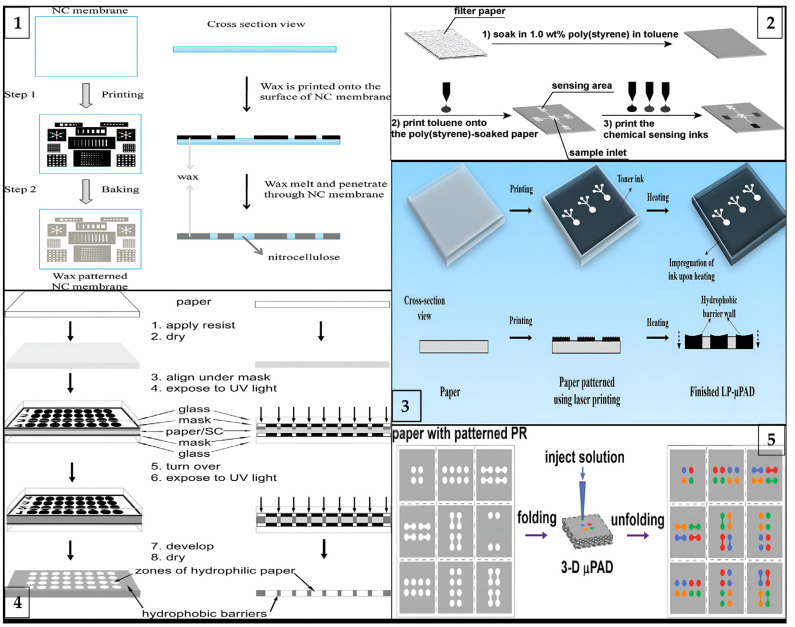
Schematic illustration of different types of fabrication processes for μPADs: (**1**) laser printing method (reprinted with permission from [41]; copyright 2019, Springer Nature); (**2**) wax printing method (reprinted with permission from [76]; copyright 2010, American Chemical Society); (**3**) photolithography method (reprinted with permission from [77]; copyright 2009, American Chemical Society); (**4**) three-dimensional origami method (reprinted with permission from [66]; copyright 2011, American Chemical Society); and (**5**) inkjet printing method (reprinted with permission from [36]; copyright 2008, American Chemical Society).

*Wax Printing.* This method concerns the formation of hydrophilic channels and barriers that characterize fluid reservoirs and reaction zones. Basic principles of wax printing technique is shown in Figure 2(1). The wax printing process is ideal for quickly producing large quantities of µPADs [30]. The main idea behind this technique is to spread melted wax on paper. Because the wax melts on the surface of the paper, it spreads quickly both vertically and horizontally. This vertical spread creates a hydrophobic barrier across the paper [31]. On the other hand, lateral spreading reduces pattern solubility and provides a wider hydrophobic barrier. These effects result in a wider hydrophobic barrier on the side where the pattern is printed than on the back side [29,32]. Briefly, wax patterns can be created using either a wax printer or a pen. The final step is heating in an oven or hot plate to allow the wax to penetrate the paper and form a hydrophobic barrier [33].

*Inkjet Printing.* Inkjet printers are a widely used technology in the commercial market. As a result of its popularity, inkjet printing is a low-cost technique that allows the production of paper-based biosensors with important advantages. For illustrative purposes, a paper-based microfluidic device can be produced using even a low-budget printer found in offices. Besides these advantages, the requirement for solvent treatment of paper limits their usage [34]. Therefore, in these printers, many researchers have modified the inks to fabricate μPADs and simplify the mass production process [32]. Hence, the paper is patterned using a commercial inkjet printer in which the ink is replaced by solvent. This solvent can be resin [35], polystyrene [36], or polyacrylate [37]. Printing these hydrophobic solvents on paper allows for the creation of easily controlled hydrophobic patterns. The method using polystyrene as solvent is briefly shown in Figure 2(2). In addition to creating patterns in the desired shapes, inks can be used to create specific channels for different chemicals by adding different cartridges to the printer. Thus, many substances can be analyzed on a single sheet of paper. Wang et al. described a sustainable technique for the fabrication of μPADs using hydrophobic sol-gel with inkjet printing or base etching [38].

*Laser Printing.* The laser printing method includes the use of a laser printer that can print with hydrophobic inks, and much of the procedure is similar to wax printing. In fact, laser printing can be considered an updated version of wax printing for modern times [39]. They are a new generation of wax printers, using ink cartridges instead of wax cartridges in the printer [40]. Printing directly collects hydrophobic ink automatically, eliminating the need for human intervention. As a result, laser printing has emerged as a method that significantly reduces production costs and complexity as shown in Figure 2(3) [41]. This technique uses ink cartridges to print toner onto one side of the paper. It is then heated to melt and spread the toner ingredients onto the paper. Even though this technique needs higher temperatures and longer processing times, laser printers are more common and cheaper than solid ink printers.

*Plotting.* Plotters or templates are used for the patterns to be created in the plotting technique. For instance, hydrophobic inks such as polydimethylsiloxane (PDMS) dissolved in hexane are used to print the pattern on paper [31]. Considering their benefits, this technique allows production in a cheap, and flexible structure [42]. Plotting does not require any heating step as the ink penetrates during drawing. On the other hand, having low resolution and unstable liquid layers can be considered a disadvantage of the plotting technique. 

#### 3.2.2. Masking Methods

*Photolithography.* This is a delicate form of custom surface fabrication in which the interface of a wafer is coated with a photosensitive polymer known as a photoresist. The first paper-based microfluidic fabricated using photolithography was reported by Whitesides and his group in 2007 [43]. They used a hydrophobic photoresist, SU-8 polymer. The main advantage of this method is its high resolution, but it requires expensive equipment. Moreover, the production stages are very complicated, which explains why it is not a widely used method nowadays. On the other hand, many studies have been performed to reduce the high cost of photolithography. Martinez et al. formulated an epoxy-based negative photoresist to reduce complex production steps and costs [44]. In a typical process, the paper is first impregnated with the photoresist precursor; when exposed to UV in a certain order, the photoresist can selectively crosslink in the paper and the uncured monomers are then washed away. In this way, the areas occupied by the photoresist become hydrophobic, while the remaining areas still remain hydrophilic, allowing the transport of liquids [45].Photolithography method is briefly illustrated in Figure 2(4) below.

*Wax Dipping.* In this method, metal molds prepared in desired shapes with the help of a device are used to create hydrophobic barriers to be drawn on paper. The mold is placed on the paper and strong magnets are used to ensure that it does not move in order not to distort the pattern. The whole assembly is then dipped in molten wax. The magnets used here are strong enough to prevent the molten wax from seeping under the metallic mold. Once completely covered with wax, the prepared apparatus is removed, and the excess wax is scraped off. The metal molds are removed from the paper and the paper is heated to about 40 °C. This allows the wax to melt and completely penetrate the paper [46].

*Plasma Treatment.* This technique provides a method for making μPADs using plasma treatment. The paper is first made hydrophobic and then selectively hydrophilized with a metal mask by exposure to plasma [47]. Using this method, switches and filters can be built directly into the circuit. In this method, the paper is first immersed in an AKD–heptane solution. Then, the paper is placed in a fume cupboard to remove the heptane. After the solvent is removed from the paper surface, the paper is placed in an oven at a certain temperature to cure the AKD. To obtain the desired pattern, the paper is placed between metal stamps as in a sandwich. The areas exposed to the plasma will become hydrophobic, forming the channels. Metal stamps are created by mechanically cutting steel sheets [48].

#### 3.2.3. Design-Pattern Methods

*Screen Printing.* In this technique, a mesh sieve is used to selectively apply wax to the paper to form the boundaries of the fluid channel. A hydrophobic ink or wax is printed from a patterned screen onto the paper in the desired pattern. The advantage of this technique is that it has a wider range of inks. The hydrophobic material can be wax, PDMS, poly(methyl methacrylate) (PMMA), polystyrene, etc. [49].

*Stamping.* Stamping is a widely used method for fabricating microfluidics. Its ease and convenience have led many researchers to try to produce µPADs using different types of stamps and inks [50]. Stamps patterned from various materials are used to contact hydrophobic ink in a single step, and then the ink is transferred to paper. Stamping can be performed using methods such as PDMS stamping and wax stamping. If PDMS is used for the hydrophobic barrier, a rubber stamp of the desired shape is dipped into the PDMS solution. Then, the stamp is printed on the paper. Thus, the PDMS with the shape on the stamp will be transferred to the paper. The process is then completed by drying in the oven [51].

#### 3.2.4. Cutting/Shaping Methods

This method uses a laser cutter or x-y cutting plotter to remove material from the paper substrate. This creates an air gap between the channel and the surrounding paper without the use of any chemicals or processes. This technique provides a one-step and very fast process [52]. The laser cutting method is to identify channels in a sheet of paper by cutting the outline of the desired pattern using a knife or a laser. The solvent flow direction is thus controlled by the edges [53]. While it is a simple method that does not require chemicals, one of the disadvantages is that the cutting process can result in a collision or tear. To avoid damage, many researchers have worked to perform the operation after loading paper onto a backing. Fenton et al. created a microfluidic device by using a polyester backing [54].

**Table 1:** µPAD manufacturing techniques to detect different types of tumor biomarkers.

Analyte	Matrix	Fabrication Technique	Substrate	Signal Detection Technique	Reference
AFP, CEA CA125 CA153	Serum	Photolithography	Chromatography Paper	Electrochemical	[55]
CEA	Serum	Photolithography	Chromatography Paper	Chemiluminescence	[56]
CEA, NSE	Serum	Wax and Screen Printing	Chromatography Paper	Electrochemical	[57]
CEA	Serum	Wax Printing	Filter Paper	Electrochemical	[58]
CEA, PSA	Serum	Wax Printing	Chromatography Paper	Electrochemiluminescence	[59]
CEA, PSA	Serum	Wax Printing	Chromatography Paper	Fluorimetry	[60]
Citrate	Urine	Laser Cut	Chromatography Paper	Colorimetric	[61]
CA 15.3	Plasma	Inkjet Printing	Photographic Paper	Chronoamperometry	[62]
CEA	Serum	Manual		Colorimetric	[63]
MCF-7	Tumor Cell	Wax Printing	Chromatography Paper	Electrochemiluminescence	[64]
PSA	Serum	Wax Printing	Chromatography Paper	Voltammetry	[62]

### 3.3. Three-Dimensional (3D) Microfluidics

Researchers have developed 3D manufacturing methods for use in multi-step chemical reactions and processes requiring multiple pre-processing. This allows all steps to take place on a single chip and increases the number of analyses [39]. Three-dimensional µPADs have stereo channels, making them superior to 2D µPADs. 

The analytical principles of 2D and 3D μPADs were first described by Whitesides et al. [65]. Hence, the first steps have been taken toward a multifunctional device without increasing the device size. Three-dimensional μPADs that allow liquids to transfer in all three dimensions are made by stacking two or more patterned papers. As a result, the fluid moves along the stacked layers on the paper in the x, y, and z planes. This reduces turnaround time compared to the 2D μPAD. This means that the 3D device can rapidly deliver samples from a single inlet to dozens of test sites by passing liquid vertically and horizontally through layers of patterned paper [65]. Researchers have published approaches such as origami and stacking for the fabrication of 3D μPADs. The basic fabrication principle of the 3D origami method is shown in Figure 2(5) Liu and Crooks reported a method for 3D devices based on the principles of origami (folding) [66]. Martinez et al. also reported the fabrication of a 3D device using the stacking method [65]. This device had the ability to detect glucose and protein, as well as a control zone.

### 3.4. Paper Pretreatment and Modification for Biofunctionality

Nitrocellulose membranes or cellulose-based papers are used in the production of μPADs. Nitrocellulose membranes are porous and hydrophobic in nature. As a result of their hydrophobicity, non-specific binding occurs and this reduces the sensitivity of the developed device [67]. Therefore, the shelf life of uncoated devices is lower compared to functionalized and/or biofunctionalized ones. There are a few options for biofunctionalizing the surface of the paper used as substrate in microfluidic devices. Hence, biomolecules are usually attached to the paper by passive adsorption and electrostatic interaction [68,69]. To biofunctionalize the surface of the paper, biomolecules are usually bound to the membrane by adsorption. It is assumed that the biomolecules will interact with nitrocellulose membranes containing hydrophobic carbon, causing the physical deposition of biomolecules. Another method of biofunctionalization is based on electrostatic interaction. In this case, it is the electrostatic interaction of the dipoles of the biomolecule with nitrated groups that have dipole forces. Passive adsorption and electrostatic interactions can be performed using photoresist agents. These agents include polymers such as polydimethylsiloxane and methacrylate. Using all these methods, it is possible to change the wetting behavior of the paper by completely covering the membrane, including its pores [3,23].

In μPADs, a binding or reaction probe is first immobilized on the surface to be functionalized. This immobilization greatly affects the reliability and sensitivity of the developed device. As a result, surface immobilization is a primary design and performance consideration. In general, μPADs require the use of a ‘probe’ on the paper surface to capture native and post-translational proteins. These probes are biomolecules such as aptamers, proteins, antibodies, and antigens, and are immobilized inside the microchannels. Avidin–biotin, protein A/G-antibody, genetically engineered protein affinity ligands, DNA hybridization, and aptamers are probes used in microfluidic devices. One of the most widely used immobilization partners is the combination of avidin and biotin. Avidin binds to biotin through highly strong non-covalent interaction. The binding interaction is rapid and almost insensitive to pH, temperature, proteolysis, and denaturing agents [70]. A disadvantage of using the avidin–biotin system is the high cost of the protein-binding reagent. NHS (N-hydroxysuccinimide) ester is a popular commercial biotinylating reagent that enables the covalent binding of protein amine groups with biotin. Due to the popularity of the avidin–biotin immobilization strategy, streptavidin-coated polystyrene, agarose, and glass beads are commercially available. These beads are widely used in microfluidic experiments [71]. On the other hand, regardless of its high cost, variable affinity, and short shelf life, an antibody is a ubiquitous biomolecule for immobilizing proteins. The widespread use of antibodies is due to their exceptional specificity toward the binding partner [72]. Aptamers are oligonucleotide bioaffinity capture reagents of great interest. Aptamers that show the highest affinity for a target protein are synthetically selected. Aptamers are smaller than antibodies, so capture agents can be coated on surfaces at higher densities to provide a high binding capacity for target proteins. Arenal et al. reported an EELS-STEM (spatially resolved electron energy loss spectroscopy (SR-EELS) using a scanning transmission electron microscope (STEM)) system to identify and localize functional biomacromolecules at a nanometric resolution on the surface of a PG/Ab functionalized magnetic nanoparticle [73]. This system allows the direct identification and study of biological components (protein G and anti-HRP antibody) in complex bio-nanocarriers relevant for biomedical applications.

The choice of the material as the surface for the immobilization of the respective receptors depends on the application covered and the fabrication method of the devices. The immobilization of receptors is influenced by several factors such as the chemical and physical properties of the surface, the immobilization method, and chemical conditions, such as pH, temperature, buffer solution used, the properties of the molecules to be bound and the desired position, orientation, and density of the receptors on the surface. Immobilization can be carried out covalently and non-covalently. Non-covalent immobilization is based on bioaffinity and physisorption. Physisorption is a physical encapsulation and entrapment of receptors without involving complex chemistry or reagents. This method is dependent on environmental conditions as well as weak intermolecular bonds such as electrostatics and van der Waals. On the other hand, bioaffinity is based on binding phenomena that can be found in nature. Thus, it provides specific and directional control. This type of binding is often combined with physisorption and covalent bonding. Commonly used partners for bioaffinity interaction are avidin/biotin and streptavidin/biotin. For example, to perform DNA hybridization, DNA and proteins are often covalently linked or attached using a biotin–streptavidin linkage. These promising ligands offer many advantages, such as an excellent binding affinity for analytes, a simple chemical modification using binding molecules and dyes, and high-density functionalization of surfaces. Covalent immobilization is frequently used in μPADs. It should be preferred to physisorption, especially in cases requiring more stable and irreversible immobilization of biomolecules. However, covalent immobilization leads to the random orientation of the receptors, which may affect their biological activity and ability to bind analytes from the solution. Apart from the immobilization method mentioned here, there are many other methods. For example, Parracino et al. reported the creation of prostate-specific antigen (PSA) and Fab anti-PSA biosensor arrays using UV light-assisted molecular immobilization (LAMI) for the detection and quantification of PSA, a cancer marker, while presenting a technique as powerful as immobilization [74]. An in-depth description of each method can be found in the extensive reviews by Kim and Herr [75]. 

All these factors are important to consider because they can strongly influence the sensitivity and limit of detection (LOD) of the resulting device. Not only do the receptors need to be functional immediately after immobilization, but they also need to have a shelf life proportional to the storage duration of the devices.

## 4. Signal Detection Techniques

In μPADs, signal detection techniques are generally categorized under two main headings: electrochemical and optical. While electrochemical devices measure through electrodes, optical sensors use visible measurement methods, such as colorimetry, fluorescence, and chemiluminescence [78,79]. In this chapter, the use of various signal measurement mechanisms in μPADs for the detection of cancer biomarkers will be discussed.

### 4.1. Electrochemical

In electrochemistry, signal generation depends on the changes that occur as a result of oxidation and reduction reactions of biomaterials immobilized on the surface of electrodes [80]. When electrochemical transducers are integrated with microfluidic systems, miniaturized, multi-measurement, and highly sensitive tests with low detection limits are developed and contribute to POC technologies with powerful sensor features. [2,80]. Due to these advantageous properties, there are many applications in non-invasive aptamer-based methods for monitoring organ responses in dose-dependent drug therapies (organ-on-a-chip models), real-time biomolecule diagnostics without the need for laboratory equipment (lab-on-a-chip technology), and food and environmental safety control. Moreover, microfluidic electrochemical methods based on microfluidic fuel cells, which can power small devices for at least 1 h, when combined with paper-based methods, help to create simple, portable, and innovative devices [81,82,83,84,85].

These approaches have recently been applied to produce POC devices for cancer biomarker diagnostics. In order to improve the electrochemical measurement performance, Wang et al. modified multi-walled carbon nanotubes on a µPAD and simultaneously detected cancer antigen 125 (CA125) and carcinoembryonic antigen (CEA) tumor markers from serum samples using a 3D platform generated by horseradish peroxidase (HRP)-labeled signal antibodies. The analytical performance of the assay was compared with the commercially available electrochemiluminescence method. Relative errors were found to be less than 5.8% for CA125 and less than 6.5% for CEA, and an acceptable agreement was found between the two methods [86].

**Figure 3:**
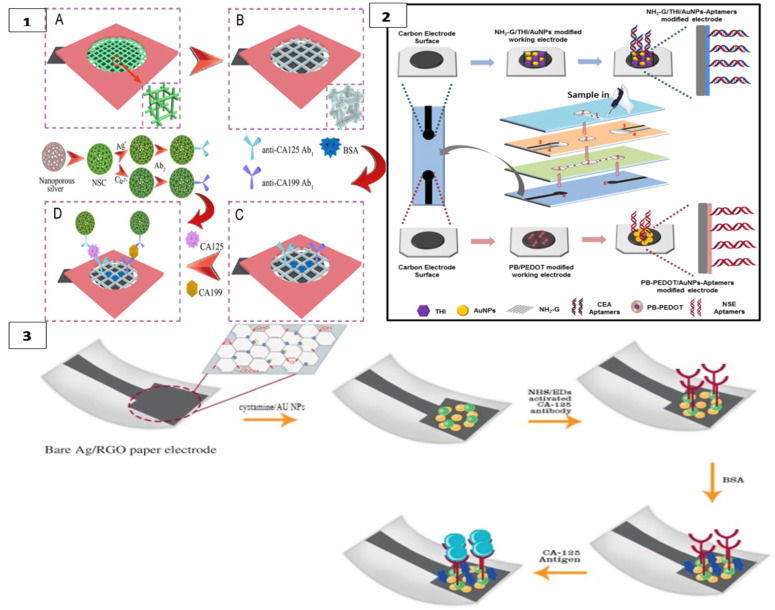
(**1**) Cuboid silver-paper electrode-based multiplex electrochemical origami immunodevice (A) Bare paper working electrode (PWE); (B) Cubic silver modified paper working electrode (CS-PWE); (C) after immobilization with Ab1s and blocking; and (D) after capturing, washing and incubating with the designed labels. (reprinted with permission from [87]; copyright 2014, Elsevier); (**2**) label-free microfluidic paper-based electrochemical aptasensor (reprinted with permission from [57]; copyright 2019, Elsevier); (**3**) paper-based immunosensing of ovarian cancer tumor protein CA 125 using nano-ink (reprinted with permission from [88]; copyright 2019, Elsevier).

CA125 and CA199 were detected by square wave voltammetry scanning on another platform constructed with a cubic silver modified paper working electrode (CS-PWE) using nanoporous silver chitosan (NSC) coated with different metal ions as a label, as shown in Figure 3(1).

In this 3D multiplex microfluidic study, the measured peaks increased with a higher concentration of the target analyte. Copper and silver metal ions used as labels were suggested to enhance the peaks. Detection limits of 0.02 and 0.04 mU/mL^−1^ were found for CA125 and CA199, respectively. Since the threshold value of these measurements with real samples was 35 U mL^−1^ for both analytes, a significant result was obtained for POCT [87]. Li et al. detected CEA in their first study using a method similar to this one [89].

In another study, CEA and NSE biomarkers were simultaneously detected for early diagnosis of lung cancer using a label-free electrochemical method. Amino functional graphene (NG)–thionine (THI)–gold nanoparticles (AuNPs) and Prussian blue (PB)–poly (3,4-ethylenedioxythiophene) (PEDOT)–AuNPs nanocomposites were modified on the surface of the working electrodes to enhance aptamer immobilization, as in Figure 3(2) The analyte was detected in the results of DPV measurement with a decrease in current with the formation of the aptamer–antigen complex on the electrode surface [57].

Bahavarnia and co-workers have developed a paper-based Ag/RGO (silver nanoparticles/reduced graphene oxide) ink on the electrode surface. The surface of these electrodes was modified with cysteamineA-functionalized gold nanoparticles (CysA/Au- NPs). Then, the CA125 antibody was immobilized on the surface (Figure 3(3)). In the presence of CA125 antigen, the current measured by chronoamperometry was significantly reduced [88]. In another study, the electrode surface was formed with graphene oxide (GO) on the paper surface and DNA aptamer was immobilized on the working electrode for the detection of prostate-specific antigen (PSA) [62]. Moreover, recently, AuNPs were used as a modifier on cellulose fibers together with MnO_2_ nanowires placed on the electrode to obtain a larger surface area. In this system, glucose oxidase was used as a label and PSA was measured with high sensitivity [90].

### 4.2. Colorimetric

Colorimetric tests are based on the monitoring of target molecules through a visible signaling agent. As a result of the colors that exist in the presence or absence of the analyte, both qualitative and quantitative measurements can be obtained. In parallel to their integration with smart devices, they are used in many areas from food safety, such as in meat, milk, and drinking water, to environmental pollution, as well as the determination of analytes from biological fluids and detection of reactive oxygen released from some cells [91,92,93,94,95,96,97,98,99,100].

Colorimetric methods usually provide qualitative measurements with visual responses but can be converted to quantitative measurements with color analysis on smart devices. Because of their easy and fast combination with technological devices, nowadays they are considered very promising tools in the diagnosis of diseases such as cancer and infections in which early diagnosis is important. For instance, in a platform using glutaraldehyde as a cross-linker to immobilize antibodies on chitosan-treated paper in the presence of an analyte, the analyte binds to immobilized antibodies according to the sandwich assay method, and the HRP-labeled antibody binds to it. When 3,3′,5,5′-tetramethylbenzidine (TMB) chromogen substrate is added, a blue color is formed in the signal zones. When a photo is taken with a phone, color analysis is applied automatically, and CEA concentration is calculated with the developed application. The platform, which was also tested with clinical samples, was able to detect CEA concentration with a 98% sensitivity [101]. With a similar method using the sandwich method, different concentrations of CEA were detected at different detection sites and quantitative determination was made by color analysis with Image J software [102]. There are also ultra-sensitive studies for the colorimetric detection of CEA as a result of the excitation of plasmonic nanoprisms (NPR) with a near-infrared spectrophotometer (NIR) and changing the temperature around the NPR. However, a device such as NIR for signal detection can be made suitable for POC technologies by making it portable and integrating it with smart devices [103].

**Figure 4:**
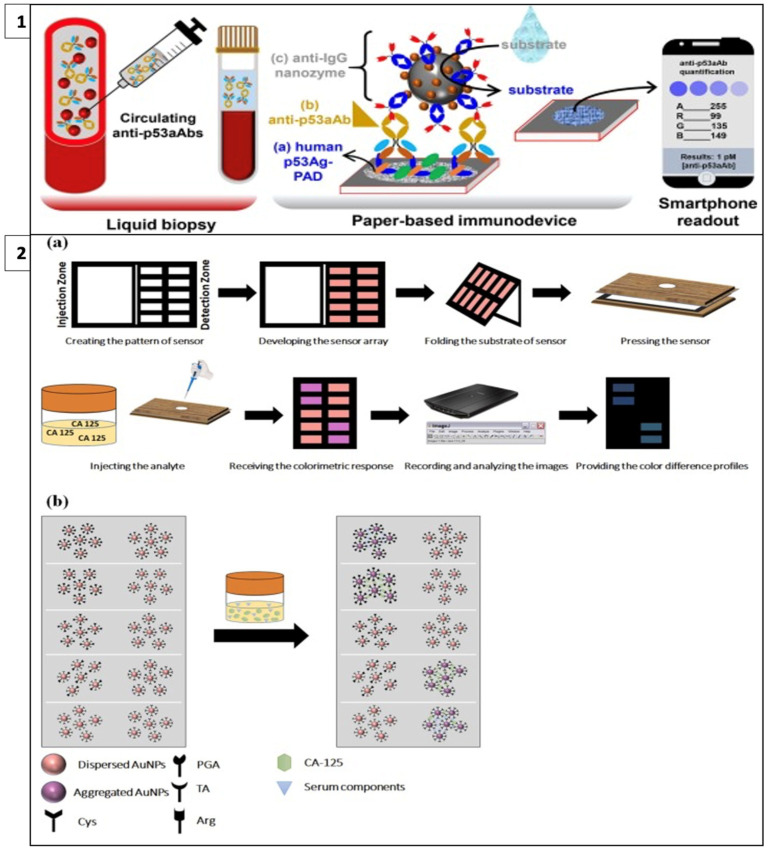
(**1**) Detection of tumor-specific anti-p53 autoantibodies (anti-p53aAbs) (reprinted with permission from [104]; copyright 2022, Elsevier); (**2**) colorimetric cancer detection system called fingerprint response model (a) fabrication and application of the proposed sensor array and (b) interaction of sensor components and CA-125 cancer marker. (reprinted with permission from [105]; copyright 2022, Elsevier).

Adeniyi and Mashazi developed a method for the detection of tumor-specific anti-p53 autoantibodies (anti-p53aAbs) that allows rapid and on-site diagnosis instead of commonly used techniques such as Western blot and ELISA. In this method, human p53 antigen immobilized on paper was used for the detection of anti-p5aAbs for antibody recognition and capture, as in Figure 4(1). Fe_3_O_4_@SiO_2_-prNH2-Au@Pd0.30NPs-anti-IgG immunoprobes were used as catalytic labels for substrate oxidation. In the presence of an analyte, blue dots were formed on the paper; these dots were photographed with a mobile phone and the results were read with color analysis software. It was observed that the color intensity increased with increasing anti-p53aAbs concentration. This method can be used as an easy and accessible alternative to liquid biopsy. [104].

Bordbar et al. have developed a platform called the fingerprint response model for a specific antigen with bi-functionalized AuNPs. This enabled the simultaneous detection of four different cancer biomarkers in serum with a prediction error of less than 6.0 U/mL. In this model, when four antigens with different chemical structures and sizes interact with nanoparticles that have a stabilized distribution at a specific detection site on paper, the repulsion between the nanoparticles decreases as steric hindrance is eliminated and the color of the receptor changes from red to purple, as in Figure 4(2). The resulting colors are then analyzed with Image J software [105].

Citrate in urine is used as an early detection biomarker of prostate cancer. In a study developed for the determination of citrate, cysteine-coated gold nanoclusters (Cys–AuNCs) catalyze the oxidation of TMB in the presence of peroxidase, producing a blue color. In the presence of citrate, citrate binds to Cys–AuNCs and inhibits color formation. Based on the decrease in color intensity, a linear relationship was obtained for the measured citrate concentrations in the range of 0.5 to 1000µM [61]. These methods led to the development of non-invasive and multiple measurements with a small sample volume.

### 4.3. Fluorescence

The fluorescence methodology relies on light emission based on the analyte concentration on the sensing surface. Excited by a beam of light, the fluorescence label moves from the fundamental energy level to a higher energy level. At this level, the molecule is unstable and emits the excess energy it absorbed as light emission and returns to the basic energy level. By measuring this light emission during this transition between energy levels, the fluorescence signal against the analyte amount is detected.

**Figure 5:**
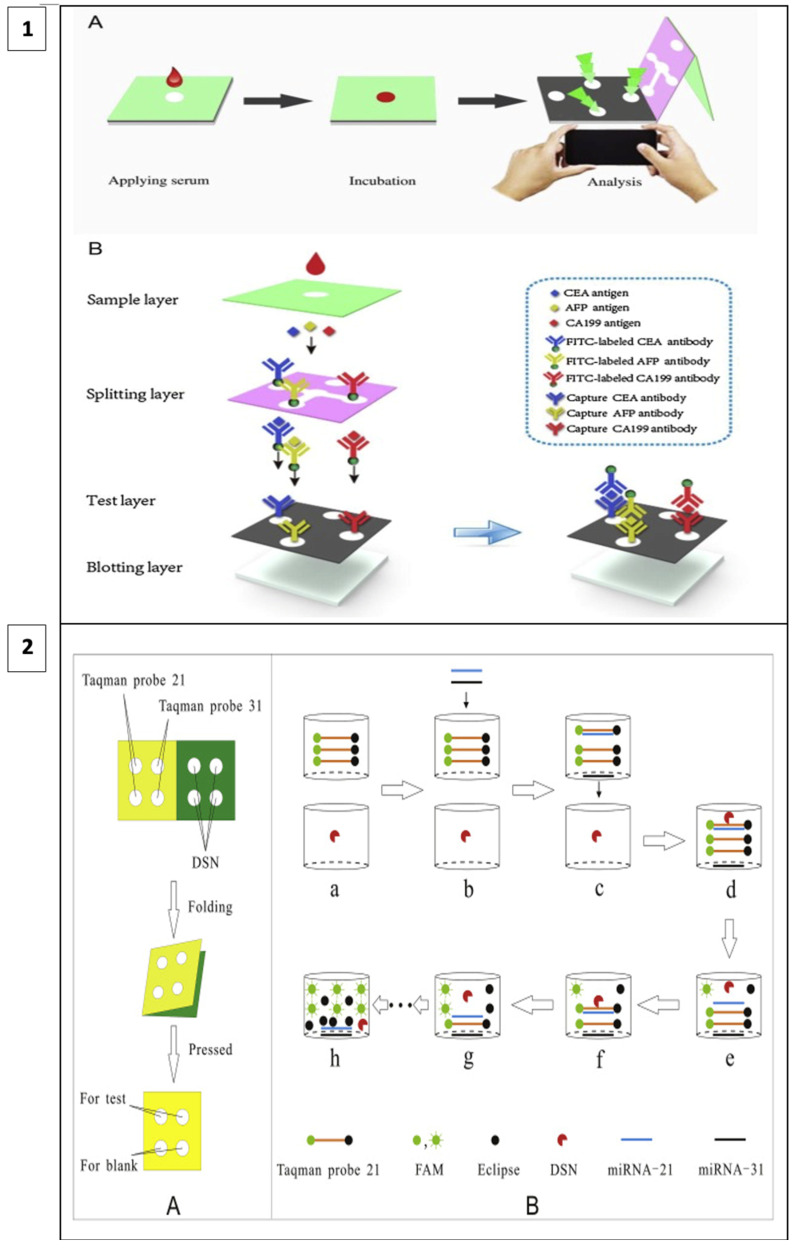
(**1**) Three-dimensional vertical flow paper-based microfluidic assay (reprinted with permission from [106]; copyright 2020, Elsevier); (**2**) miRNA detection by laser-induced fluorescence μPAD (A) The process of μPAD fabrication. (B) The process of fluorescence amplification for miRNA on μPAD. (reprinted with permission from [107]; copyright 2020, Elsevier).

When fluorescence methods are integrated with μPAD designs, the cost of fluorescence methods can be reduced, and detection thresholds can be lower than with other methods. For example, in a study including a 3D vertical flow μPAD prepared with the origami technique, fluorescence isothiocyanate-labeled antibodies were placed on the separation layer and mouse monoclonal capture antibodies on the test layer (Figure 5(1)). In this sandwich-type fluorescence immunoassay, in which CEA, alpha-fetoprotein (AFP), and CA199 are detected, fluorescence irradiation is observed at the test sites in the presence of the target. A comparison of the test results with other assay methods has shown that this method has a broader detection area, lower detection thresholds, and less time lost in terms of detecting three different analytes simultaneously [106].

In a recent study, a method utilizing duplex-specific nuclease (DNS) amplification was reported to detect miRNAs which are biomarkers of many types of cancer. DNS can selectively cut DNA strands as in Figure 5(2). When fluorescent Taqman probes (DNA strands) hybridize with target miRNAs, DNS cleaves this hybridized structure, and the fluorescent tags are released. With this method, multiple miRNA assays can be performed at the same time and the test is portable as it allows miRNAs to be dried and stored on paper [107].

In another study, aptamer-conjugated silica nanoparticles coated with quantum dots (QDs) were prepared and used for a fluorescence quenching-based measurement mechanism on a graphene oxide surface to monitor cancer cells. By utilizing the quenching effect of graphene oxide, fluorescence was quenched by Förster resonance energy transfer. Fluorescence was restored when target cells were added. With this method, MCF-7, HL-60, and K562 cells were visibly detected simultaneously with aptamer probes prepared in different colors. It was emphasized that this study is an improvable method for clinical studies [108].

Abnormally elevated levels of alpha-fetoprotein in serum can be a marker for diseases such as tumors. In a sandwich assay designed for the detection of AFP, up to 1.0 pg/mL AFP was detected with low sample volume. For the experimental design, primary antibodies were immobilized on chitosan-coated paper as capturing agent for AFP, while secondary antibodies are modified with starting DNAs that stimulate the hybridization chain reaction (HCR) to raise the fluorescence signals of AFP [109].

### 4.4. Other Techniques

μPADs have also been used in cancer studies by combining chemiluminescence and electrochemiluminescence methods. In chemiluminescence, the analyte concentration is measured based on the luminescence intensity emitted because of a chemical reaction. This method has the advantage of low background signal compared to optical sensors where absorbance and fluorescence intensities are measured as a response to the light emitted by a light source [110,111]. When the reaction that enables light emission in the chemiluminescence method is initiated by applying an electrochemical potential, this method is called the electrochemiluminescence method [112].

**Figure 6:**
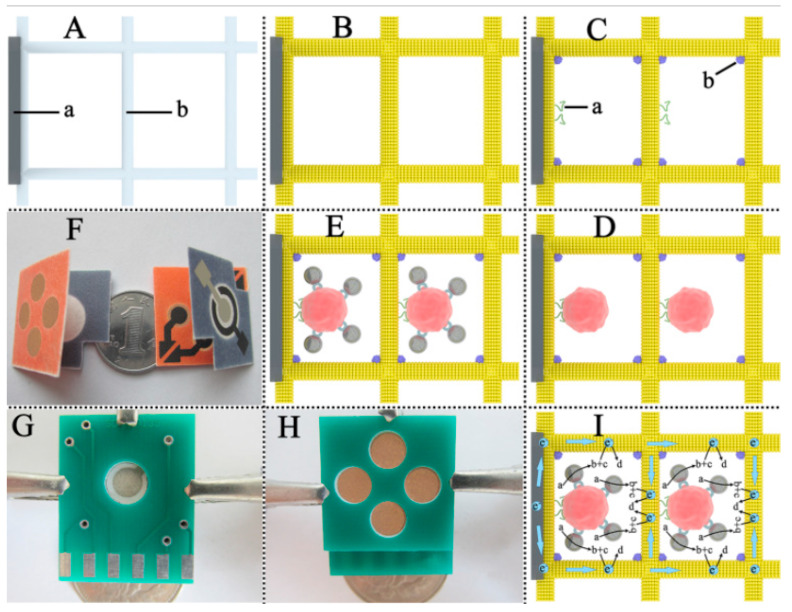
Paper-based microfluidic test system produced with origami technique for multiple cancer cell detection (**A**) Bare PCE: (a) SPCWE, (b) cellulose fibers in the paper cell zone (unprinted macroporous paper). (**B**) Growth of Au nanoparticles layer on cellulose fiber surfaces. (**C**) Immobilization of aptamer and blocking with BSA: (a) aptamer, (b) BSA. (**D**) Capture of cells. (**E**) Binding of AuPd@Con-A bioconjugates on cell surface. (**F**) Folded μ-PECLOC. (**G**) The folded μ-PECLOC was clamped between two circuit boards. (**H**) The reverse side of (**G**). (**I**) Schematic illustration of the ECL detection mechanism in Au-PCE: (a) H_2_O_2_, (b) O_2_, (c) K_2_S_2_O_8_, (d) 1(O_2_)_2_∗. (reprinted with permission from [112]; copyright 2015, Elsevier).

In a method based on a chemiluminescence origami cyto-device, as demonstrated in Figure 6, cellulose fibers were coated with AuNPs and further modified with aptamers. AuPd@Con-A (AuPd alloy nanoparticles labeled concanavalin A) bioconjugates were bound to breast adenocarcinoma cells captured by aptamers. The bioconjugate on the cell surface catalyzes the disproportionation reaction of H_2_O_2_ and the resulting O_2_ generates an electrochemiluminescence peak. This method was reported to have a lower detection limit and comparable results to other cyto-sensors [112].

Wang et al., using a method they defined as chemiluminescence ELISA, created a plate-like structure on chitosan-coated paper. They covalently immobilized capture antibodies on this structure and detected AFP, CA125, and CEA cancer antigens with the help of luminol-p-iodophenol-H_2_O_2_-HRP reaction by forming a sandwich method with HRP-labeled antibodies. In addition to being economical and practical, this method also gains attention with its storage condition of five weeks at 40 °C [113].

In a study that stands out for its production technique using recyclable polystyrene, which can be advantageous in regions where resources are limited, the luminol-H_2_O_2_ p-iodophenol system was utilized with a sandwich-type assay. CEA, AFP, and PSA cancer biomarkers were detected from serum samples. It was observed that the obtained linear results are potentially applicable for on-site diagnostic tests [114].

## 5. Application of μPADs in the Detection of Cancer Biomarkers

The early detection of cancer using disease-specific biomarkers is critical for both treatment and screening. As discussed in Section 4, biomarkers can be determined using various signal mechanism-based analysis methods. Although immunoassays are extensively used as a detection method due to their high specificity and sensitivity, some drawbacks, such as signal amplification, target labeling, and multiple washing steps, as well as the requirement for experienced technicians, create the need for these analyses to be replaced by more applicable alternative methods. μPADs overcome these operational difficulties even though their basic working principle is simple and requires small volumes because they provide a continuous process due to the reagent flow through the microfluidic channels. Although the basic operation of μPADs is simple, when other reactive solutions such as liquid samples, wash buffers, etc. are added to the cellulose membrane, the run-off of primary antibodies becomes a problem due to non-specific adsorption in these designs. The specificity of the measurement is significantly affected by the flow of the primary antibody from the test strip during analysis. The designed μPAD is intended to overcome this limitation by providing surface chemistry that will promote strong binding and provide accurate measurement of trace cancer biomarkers in the sample. For example, when the optical detection method of μPADs is investigated, the surface chemistry is first provided by adding chitosan to the surface, which provides a simple modification, and thus the analysis sensitivity is improved with more stable immobilizations with a covalent bond. For instance, Alizadeh and colleagues created a μPAD-based immunosensor for the detection of CEA. The glutaraldehyde (GA) cross-linking method was used to immobilize primary antibodies functionalized with chitosan on the paper surface. Following the incubation of the sample, a Co_2_(OH)_2_CO_3_-CeO_2_ nanocomposite functionalized with secondary antibodies was added to the sensor. The nanocomposite’s peroxidase-like activity then caused TMB oxidation in the presence of hydrogen peroxide, resulting in a color change. Sulfuric acid was then added, and the color change from blue to yellow was observed. Color can be seen with the naked eye, and quantitative results were obtained by capturing images with a smartphone and analyzing them with specialized software [63]. Wang and colleagues developed a chemiluminescence immunoassay-based PAD with an integrated magnetically actuated valve and an adjustable time controller for the rapid determination of numerous tumor markers. Photolithography was used for the patterning and fabrication of the PAD. The capture antibodies (CEA, AFP, and CA-125) were immobilized by chitosan coating and glutaraldehyde cross-linking. The time controller’s timing channel included two adjustable conductive iron bands that functioned as an electric switch. The circuit was closed, the magnetic valve was activated, and the chemiluminescence immunoassay reaction was started by connecting these bands via fluid flow. In comparison to the conventional method, the reaction based on HRP-O-phenylenediamine-H_2_O_2_ required only about 3.4 min of incubation time [56]. Timed fluid control (TFC) is a fascinating method for automating immunoassay-based PADs. TFC can be accomplished in three ways: (1) by changing the geometry of the channel, (2) by adding chemicals to generate a programmable flow delay, or (3) by using a mechanical valve to control the reaction’s state. The incorporation of superior properties of nanostructures in the biosensing process contributes to the development of functional PADs and the provision of higher analytical performance, as well as the shortage of practical applications in this field. Recent applications have clearly demonstrated that these PAD systems have the potential to be integrated into POCs (such as telemedicine and smartphone applications), and they can be improved and developed as new requirements arise. Despite the fact that research in this area is limited, this section presents important and recent strategies for the use of μPADs in cancer application [17,115] In Table 2, comparison of μPAD applications in the detection of cancer biomarkers were given in terms of target biomarker, fabrication and detection technique, surface chemistry and analytical performance parameters including linear range and LOD. 

### 5.1. Detection of Protein Biomarkers

Proteins are biomolecules found in the body that, when expressed abnormally, can be associated with a variety of diseases, including cancer. Cancer cells can produce proteins that they can release into body fluids such as blood and urine. Cancer proteins, unlike other proteins, are difficult to identify due to their low concentration. Accordingly, single protein detection or multiple protein detection using different signal-enhanced detection strategies can be used to monitor cancer detection, cancer stage, and treatment process [115].

Wang et al. created a multi-parameter paper-based electrochemical aptasensor with high sensitivity and specificity for the simultaneous detection of CEA and neuron-specific enolase (NSE) in a clinical sample. The microfluidic channels in the system were created using wax printing, while the three-electrode system was created using screen printing. Prussian blue (PB)–poly(3,4-ethylenedioxythiophene) (PEDOT)–AuNPs nanocomposites and amino functional graphene (NG)–thionine (THI)–AuNPs were produced. These nanocomposites were used not only to boost electron transfer rates, but also to replace working electrodes in the immobilization of CEA and NSE aptamers. To detect CEA and NSE in clinical samples, a fast, simple, and label-free electrochemical method was used. To validate the clinical application of the developed aptasensor, fifteen clinical serum samples provided by the Peking University Cancer Hospital were tested using a commercially available Roche electrochemiluminescence apparatus. Under optimal conditions, the multi-parameter aptasensor showed excellent linearity in the 0.01–500 ng mL-1 range for CEA (R^2^ = 0.989) and 0.05–500 ng mL^−1^ range for NSE (R^2^ = 0.944), respectively. The 3/S calculated the LOD to be 2.0 pg mL^−1^ for CEA and 10 pg mL^−1^ for NSE. The price of a paper device was calculated to be around $0.12. As a result, the presented device could offer a low-cost and portable diagnostic platform for cancer biomarkers [57].

In another study, a paper-based chip immunoassay was developed to analyze AFP using the sandwich method. To capture AFP, primary antibodies are immobilized on paper using chitosan. In secondary antibodies, altered starter DNAs can trigger a hybridization chain reaction to amplify fluorescence signals for AFP. A laser-induced fluorescence detector integration was used to detect the targets. Under ideal conditions, the limit of detection for AFP in the study was 1.0 pg/mL [109].

In contrast to traditional paper-based microfluidic analytical devices, developed innovative 3D μPADs can control fluid flow dynamics in 3D using only capillary-driven flow and no external pump. A sequential and automatic multi-step ELISA test can be performed through the 3D bridge structure by simultaneously adding the sample, washing, and enzyme–substrate solutions used in the study to the system in the 3D μPAD. Thioredoxin-1 (Trx-1) was studied as a target biomarker in this 3D μPAD study, which is a new breast cancer biomarker in the literature. The results demonstrated that the 3D μPAD provides a 0–200 ng/mL detection range for Trx-1. Sera from patients and healthy people were used to confirm that this chip is suitable for commercial use and that Trx-1 can be detected accurately in real serum samples. Validation studies using ELISA revealed significant differences for this new biomarker between healthy and sick people. As a result, it is clear that the 3D μPAD created in the real-world example is a commercially extensible platform [116].

Yang and colleagues produced fully functional tests that truly achieve the goal of POC testing. The microfluidic channel was created using wax printing, while the electrodes were created using screen printing. The researchers successfully synthesized NH_2_-G/Thi/AuNPs nanocomposites, which were then used to modify the working electrode. The working electrode had high bioactivity after modification, indicating that it could be used to detect CEA with immobilized anti-CEA. A label-free electrochemical method that avoids labeling antigens or antibodies during measurement was used to make CEA detection faster, simpler, and less expensive. The results of the experiment revealed that the limit of detection for CEA was 10 pg mL^−1^ (S/N = 3) and the correlation coefficient was 0.996 in the range of 50 pg mL^−1^ to 500 ng mL^−1^ [58].

The sensitive and selective detection of PSA, a commonly used marker in the diagnosis of prostate cancer, remains challenging. A label-free μPAD for accurate electrochemical detection is reported by Wie et al. In this study, wax printing was used to create the microfluidic channel, and screen printing was used to create the triple-electrode system. By creating AuNPs/reduced graphene oxide (rGO)/thionine (THI) nanocomposites, the working electrode surface was modified to provide immobilization of the probe DNA aptamer sequence. The excellent conductivity of AuNPs and rGO also contributes to electron transfer, while THI acts as an electrochemical mediator to communicate the biological recognition between the DNA aptamer and PSA. In the study, LOD was detected at 10 ng/mL and a linear range of 0.05 to 200 ng mL^−1^. Real samples from Shijitan Hospital were analyzed to further investigate the applicability of the developed aptasensor in clinical settings. The results of clinical serum samples obtained from Shijitan Hospital using the proposed aptasensor were compared to reference values obtained in the hospital using the electrochemiluminescence method (Roche, USA). The analytical results were generally compared with hospital reference values, with relative errors calculated to be less than 8.81%. In conclusion, the proposed aptasensor is proposed to be used to detect PSA in clinical samples [62].

Fan et al. created a wireless POCT system that includes μPADs, an electrochemical detector, and an Android smartphone for the electrochemical detection of NSE. Nanocomposites containing amino functional graphene, thionine, and AuNPs (NH_2_-G/Thi/AuNPs) were used to modify PADs. It demonstrated good linearity across the concentration range from 1.0 ng mL^−1^ to 500 ng mL^−1^. The detection results were displayed in real time on the smartphone via Bluetooth connection. For a real sample analysis, the wireless POCT system also demonstrated remarkably good agreement with the ELISA method. The wireless POCT system can primarily focus on the detection of other tumor biomarkers because of its low cost, high accuracy, and low detection limit. Leading healthcare institutions as well as nations with limited resources use it. This integration of wireless systems with various storage systems can also play a significant role in telemedicine [117].

In another study, Mohamed Shehata Draz and his colleagues published a paper–plastic microchip (PPMC). The CEA and AFP amounts in a single sample were determined using the impedimetric analysis technique with the suggested PPMC. In this study, the imprinted electrolytes were designed as semicircular while developing the PPMC, and target analytes were detected by immobilizing anti-CEA and anti-AFP to the detection regions. Impedance measurements of AFP and CEA on PPMC revealed a detection limit of 102 ng mL^−1^ for both targets when using the multiplex detection format; when using the single analyte detection format, these limits were reduced to 10 ng/mL and 1.0 ng/mL, respectively. Thus, the sensitivity of the PPMC has been improved. The flexible PPMC system, which can detect analytes and conduct impedimetric analysis, is thought to be useful for wearable technology-based cancer detection [118].

The epidermal growth factor receptor (EGFR) was detected by a label-free method with extreme sensitivity using anti-EGFR aptamers as the biorecognition component in a study reported by Wang et al. In the device, the origami concept was applied in order to decrease sampling volumes and improve usability. The nanocomposites created by attaching the aptamers to the surface with H_2_-GO/THI/AuNP were added to the working electrode surface and the surface structure was changed. The detection limit for CEA was determined as 10 pg mL^−1^ (S/N = 3), with the R^2^ of 0.996 in the range from 50 pg mL^−1^ to 500 ng mL^−1^. Analyzing sera revealed an excellent correlation with the gold-standard ELISA, demonstrating the analytical accuracy of the paper-based aptasensor [119].

Based on the origami structure, Shuai Sun et al. proposed an mPAD to detect vascular endothelial growth factor C (VEGF-C). VEGF-C in serum samples was determined using an electrochemical analysis method in this study. The flow channels were created using wax printing, while the electrodes were printed on filter paper using screen printing. The working electrode was then modified with nanocomposites of methylene blue, amino functional single-walled carbon nanotubes, and gold nanoparticles (NMB/NH2-SWCNTs/ AuNPs). Thus, an mPAD with linearity ranging from 0.01 to 100 ng mL^−1^ (R^2^ = 0.988) and a detection limit of 10μg mL^−1^ was developed by improving the analysis process. The accuracy of the suggested device was achieved by supplying real clinical samples into the proposed system. As a result, this study provides an improved platform for real-time cancer detection [120].

The silver nanoparticle-reduced graphene oxide nanocomposite (Ag/RGO) ink created by Soodabeh Hassanpour and colleagues served as the foundation for the development of an immunosensor for a paper-based system. In this procedure, a high concentration of CA15-3 antibodies was added to the system following the immobilization of cysteamine-coupled AuNPs (CysA/Au NPs), which provide signal amplification, to the surface. The CA 15-3 biomarker immunosensor’s lower limit of quantification (LLOQ) was determined to be 15U/mL, and the calibration curve was linear in the range of 15–125 U/mL [121].

A new disposable and sensitive microfluidic paper-based electrochemical immunosensor on rGO-TEPA/Au electrode materials has been developed. With a simple and disposable paper-based microfluidic channel based on SPEs/rGO-TEPA/Au, both immunochromatography and immunofiltration were achieved simultaneously. Using portable SPEs/rGO-TEPA/Au and a simple μPAD, HRP-labeled signal antibodies (Ab2) and co-fixed gold nanorods were investigated as tracers for square wave voltammetry (SWV) detection. Due to the performance of HRP-GNRs-Ab2 captured at the detection site under optimal conditions, the system has wide linear ranges (0.01 ng mL^−1^–100.0 ng mL^−1^) with a detection limit of 0.005 ng mL^−1^ by using AFP as the model analyte [122].

Based on the origami principle, Yucui Jiao and colleagues created a new 3D vertical flow paper-based device (3VPD) coupled with fluorescent immunoassay that can simultaneously detect several cancer biomarkers. The device is composed of three layers: (1) sample, (2) separation, and (3) test layers so that a vertical flow immunoassay was fabricated. Fluorescent isothiocyanate (FITC)-labeled antibodies are coated on the well layer in this assay, while mouse monoclonal capture antibodies are functionalized on the test layer. The device detected three cancer biomarkers, CEA, AFP, and CA199, with detection limits of 0.03 ng/mL, 0.05 ng/mL, and 0.09, respectively [106]. It created a strategy for the instantaneous detection of multiple analytes as well as a low-cost and sensitive method for comprehensive clinical diagnosis using a single test.

Bo Dai at all. developed a flux-adaptive, self-contained microfluidic platform that includes a serological analysis platform (SAP) where CLIAs for multiple teardrop-shaped biomarkers can be run concurrently. The platform is designed to allow a low flow of sample liquid to pass through the reaction areas while speeding up the reaction as it moves between the areas. In addition, a small device for chemiluminescence detection and signal analysis has been developed. Four colorectal cancer biomarkers were immobilized to the reaction sites in the design. As a result, the microdevice added to the developed platform was used to analyze four different biomarkers at the same time. It ended up taking approximately 20 min to complete the test. CEA, AFP, CA125, and CA19-9 have detection limits of 0.89 ng mL^−1^, 1.72 ngmL^−1^, 3.62 UmL^−1^, and 1.05 U mL^−1^, respectively. Results from laboratory testing using ECLIAs based on commercial testing kits and measured using a commercial immunoassay analyzer were compared to those obtained from testing on the microfluidic platform. The calculation coefficient for the linear regression being greater than 0.9990 indicates a highly linear correlation between the SAP and the commercial immunoassay analyzer. The study in question demonstrated that the self-contained and adaptable microfluidic platform can be developed for a wide range of applications in POC-based disease health monitoring [123].

### 5.2. Detection of Nucleic Acid Biomarkers (Circulating Tumor DNA (ctDNA) and microRNA (miRNA))

The use of biofluids such as blood, saliva, and urine for cancer detection has emerged as a revolutionary technique for diagnosis and prognosis. Compared to traditional solid tissue-based biopsy tests, liquid biopsy has the advantage of being non-invasive. Therefore, it is an ideal and promising technique for monitoring and tracking cancer progression and genetic abnormalities. Liquid biopsy is a simple examination of CTCs as well as circulating tumor-derived material such as ctDNA, cfmiRNAs, and extracellular vehicles. Important information is obtained through evaluation using invasive procedures [124].

CTCs are cancer cells that have separated from the primary tumor and entered the blood system. Because CTCs are rare and highly inhomogeneous, only a small amount of sample is produced during the enrichment stage of CTC analysis. Several technologies based on CTC biophysical and biological characteristics have been developed to differentiate these cells from the background and enrich them for successive molecular or image processing analysis [124].

Exosomes are subunits of extracellular vesicles (EVs) that are essential for intercellular communication and transport of molecules from donor to recipient cells. According to recent research, exosomes have a high potential for use as novel biomarkers in liquid biopsy due to their abundance in body fluids and involvement in a variety of physiological and pathological processes [125,126].

Cell-free DNA (cfDNA), which is primarily released by hematopoietic cells, has been found for both physiological and pathological states and is currently widely used in prenatal diagnosis [124].

Small RNAs, also referred to as small noncoding regulatory RNAs, play an important function in regulating gene expression. Small RNAs are classified into three types based on their biogenesis and cellular roles: siRNAs, piRNAs, and miRNAs. miRNAs, the best understood of the three classes, have been shown to regulate at least 30% of human genes. Moreover, abnormal miRNA expression is linked to a variety of diseases, including neurodegenerative disorders, cardiovascular disease, and, most noticeably, human cancer. Furthermore, the expression of several miRNA species is altered during the progression of cancer. These specific-expression miRNAs have been identified as promising biomarkers in diagnostic procedures because of their clinical significance [127].

In a study, Xiaoyu Cai and co-workers developed and applied a microfluidic paper-based laser-induced fluorescence sensor based on duplex-specific nuclease (DSN) amplification to detect miRNAs in cancer cells in a selective and sensitive manner. DSN and Taqman probes were preserved in the circles of the folded paper chip under ideal conditions. When miRNA solution is added, the DSN can cyclically digest hybrids of miRNAs and Taqman probes, causing fluorescence signal amplification. Finally, the method was used to detect miRNA-21 and miRNA-31 in cancer cell lysates from A549, HeLa, and hepatocyte LO_2_. MiRNA-21 and miRNA-31 were detected in A549 and HeLa cells. Despite the amplification process, the analysis time was less than 40 min, and it detects very low levels of analytes [107].

Liang et al. present a fluorescent and visual method for multi-monitoring cancer cells in μPADs using GO-based aptameric biosensors. The design was based on the exceptional extinguishing capacity of GO in this method. Aptamers labeled with QD-coated mesoporous silica nanoparticles can be adsorbed on the surface of GO in a flexible single-stranded state, and the fluorescence is quenched via Förster resonance energy transfer, followed by fluorescence regeneration when target cells are added. The most important characteristic of the system was that it could be identified to multiplexed monitoring at the same excitation wavelength, making multiple detections much easier than methods using traditional organic dyes [108].

Huaping Deng et al. founded a portable and usable integrated paper fluid chip device that enables in situ HP-EXPAR amplification and optical sensing. It was created in a multi-layer format using simple paper folding. MiRNAs 155 and 21 were chosen as targets to test the performance of the platform, which used QDs as signal tags. At 90 min, the experiments yielded a satisfactory detection limit of 3 × 10^6^ copies [128].

Neda Fakhri and colleagues worked on the design of a Y-shaped μPAD. They reported that the presented method is the first paper colorimetric miRNA-21 assay based on nanocluster catalytic activity. Due to the peroxidase mimetic activity of DNA-Ag/Pt NCs, this new paper-based biosensor was created to detect sub-micromolar concentrations of miRNA-21. The detection mechanism is based on the inhibitory effect of miRNA-21 on the activity of the formed nanocluster. The system was tested on a human urine sample and the colorimetric method proved to be sufficiently accurate [61].

In a different study, researchers developed a microfluidic paper-based fluorescent biosensor in which the T-shaped duplex structure was obtained by dynamically self-assembling for the quantitative detection of miRNA and folate receptor (FR). Layered MnO_2_ nanolayers exhibiting fluorophore quenching ability were synthesized. The synthesized construct and fluorophore-labeled ssDNAs were used in the biosensor design. This recently developed biosensor has a relatively low detection limit of 0.0033 fM and could detect miRNA-21 sensitively from 0.01 to 5.0 fM. Additionally, the current sensing system has a detection limit of 0.667 ng/mL and can detect FR in the range from 2.0 to 30.0 ng/mL [129].

Liquid biopsies are a non-invasive and easily applicable technique. However, analysis using different steps such as ultracentrifugation, immunomagnetic beads, or commercial kits requires time and money. Recently developed microfluidic paper-based platforms can effectively separate and detect biomarkers from liquid biopsies with higher sensitivity than conventional methods. They can also lead to detection in potential POC applications.

**Table 2:** Summary of microfluidic paper-based biosensor applications in the detection of cancer biomarkers using various analysis methods in the literature.

Analyte	Cancer Types	Bioreceptor	Fabrication Technique	Detection Technique/Signal	Surface Chemistry	Linear Range	LOD	Reference
CEA and NSE	Lung	CEA and NSE aptamers	Wax printing and screen printing	Label-free electrochemical detection/DPV	Amino functional graphene (NG)–Thionin (THI)–gold nanoparticles (AuNPs) and Prussian blue (PB)–poly (3,4-ethylenedioxythiophene) (PEDOT)–AuNPs nanocomposites	Linearity in ranges of 0.01–500 ng mL^−1^ for CEA (R2 = 0.989) and 0.05–500 ng mL^−1^ for NSE (R2 = 0.944),	2.0 pg mL^−1^ for CEA and 10 pg mL^−1^ for NSE.	[57]
CEA, AFP, CA125, and CA19-9	Colorectal cancer	Anti-CEA, AFP, CA125, and CA19-9 Antibody	photolithography	Colorimetric method/chemiluminescent	Secondary antibody labeled with HRP, Luminol	-	0.89 ng mL^−1^ for CEA, 1.72 ng mL^−1^ for AFP, 3.62 U mL^−1^ for CA125 and 1.05 U mL^−1^ for CA19-9	[123]
CEA	Lung and other	Anti-CEA Antibody	Wax printing and screen printing	Label-free electrochemical detection/DPV	NH2-G/Thi/AuNPs nanocomposites	50 pg mL^−1^ to 500 ng mL^−1^	10 pg mL^−1^	[58]
AFP	Liver	Anti-AFP antibodies	Photolithography	Labelled electrochemical detection/SWV	rGO-TEPA/Au nanocomposite	0.01–100.0 ng mL^−1^	0.005 ng mL^−1^	[122]
EGFR	Lung and other	Anti-EGRF aptamer	Wax printing and screen printing	Label-free electrochemical detection/DPV	NH2-GO/THI/AuNP nanocomposite	0.05 to 200 ngmL^−1^ (R2 = 0.989)	5.0 pgmL^−1^	[122]
NSE	Lung	Anti-AFP antibodies	Wax printing and screen printing	Wireless point-of-care testing (POCT) system with electrochemical/DPV	NH2-G/Thi/AuNPs nanocomposite	1.0 ng mL^−1^ to 500 ng mL^−1^	10 pg mL^−1^	[117]
VEGF-C	-	Anti-VEGF antibody	Wax printing and screen printing	Label-free electrochemical detection/DPV and CV	The NMB/SWCNT/AuNPs three-in-one nanocomplex	0.01–100 ng/mL	10 pg/mL	[120]
PSA	Prostate	Anti-PSA aptamer	Wax printing and screen printing	Label-free electrochemical/DPV	AuNPs/rGO/THI nanocomposites	0.05 to 200 ng mL^−1^	10 pg mL^−1^	[62]
CA 15-3	Breast	Anti-CA 15-3 antibodies	Inkjet printing / electrodeposition	Electrochemical detection/ChA	Ag/RGO nano-ink and CysA/Au NPs	15–125 U/mL	15 U/mL	[121]
CA 125	Ovarian	Anti-CA 125 antibody	Inkjet printing/electrodeposition	Electrochemical detection/ChA	Ag/RGO nano-ink and CysA/Au NPs	0.78–400 U/mL.	0.78 U/mL	[88]
AFP and CEA	Hepatocellular carcinoma and Colorectal	Anti-CEA and AFP antibody	layer-by-layer assembly and screen-printing	Electrochemical detection/impedimetric	-	-	102 ng mL^−1^	[118]
Cancer cells of A549 and HeLa	Lung and Cervical	MiRNA-21 and miRNA-3	Wax printing	Colorimetric method/laser-induced fluorescence	Taqman probes	-	0.20 ve 0.50 fM	[107]
MCF-7, HL-60, and K562 cancer cells	Breast and leukemia	MSNs/QDs-labeled aptamers	Wax patterning	Colorimetric method/laser-induced fluorescence	QDs, MSNs/QDs and MSNs/QDs–DNA	180 to 8 × 107, 210 to 7 × 107, 200 to 7 × 107cells mL^−1^	6270 and 65 cells mL^−1^	[108]
AFP	Liver, ovaries, or testicles	Primary antibodies (Ab1) and secondary antibodies (Ab2)	Wax patterning	Colorimetric method with LIF detection/laser-induced fluorescence	Hairpin strand-FAM	2.5–1000 pg/mL	1.0 pg/mL.	[109]
Trx-1	Breast	Anti-Trx-1 antibody-conjugated with HRP	Stacking layers/cutting	Colorimetric method/optic	AgNP and Teflon ink	0–200 ng/mL	-	[116]
miRNA 155 (miR-155) and 21 (miR-21)	-	Nucleic Acid Sequences	Wax and screen printing	Colorimetric method/fluorescence	QD-labeled probes, EXPAR template of miR-21, EXPAR template of miR-150.	3 × 10^5^ to 3 × 10^8^ copies	3 × 10^6^ copies	[128]
miRNA-21 (human urine sample)	-	ssDNA template sequence	Cutting	Colorimetric method/optic	DNA–Ag/Pt NCs	1.0–700 pM	0.6 pM.	[130]
miRNA-21 and FR	Breast	Hairpin DNA Sequences	Wax printing	Colorimetric method/fluorescence	Au nanoflowers (AuFLs) and MnO_2_ nanosheets	For miRNA-21 0.01 to 5.0 fM For FR 2.0 to 30 ng/mL	For miRNA: 0.0033 fM; for FR: 0.667 ng/mL	[129]
CEA	-	Anti-CEA antibodies	Photoresist-coated	Colorimetric method/chemiluminescent	Fluorescein isothiocyanate (FITC)-labeled CEA antibody	1.0–80 ng mL^−1^	-	[56]

## 6. Smartphone Diagnosis and Telemedicine

POC devices are of increasing importance in health, environmental, and food safety analysis. They are rapidly growing as a potential alternative to traditional laboratory-based diagnostic tests, especially in resource-limited regions where the availability of medical equipment is economically constrained. Combined with biosensors and other devices, they can offer high accuracy and precision for medical testing. Moreover, the development of smartphones in recent years has greatly influenced the development of smartphone-based diagnostic kits, with mass production and related research showing promising progress [131,132]. Smartphone-based devices are needed to detect, read, analyze, transfer, and display results. Alizadeh et. al. fabricated a novel microfluidic paper-based immunosensor for the colorimetric determination of CEA [63]. In another study, Ulep et. al. developed a dual-layer paper microfluidic chip as an alternative to detect ROR1+ (receptor tyrosine-like orphan receptor one) cancer cells from the undiluted and untreated buffy coat blood samples [133]. In a different wireless POC system, Fan and his team developed an electrochemical μPAD and smartphone application to measure the neuron-specific enolase which had significance for small cell lung cancer diagnosis [117].

Most systems for the detection of cancer biomarkers are suitable for centralized clinics and not for POC diagnostics. The increasing ubiquity of smartphones, especially the increase in the quality of high-resolution imaging sensors, microphones, and ambient light sensors in recent years, makes them an attractive technology for enabling remote monitoring of patients. For cancer, where early detection and monitoring of the course of the disease is important, it is of great importance to turn to smartphone-based devices for biomarker detection. However, current applications in cancer diseases require frequent visits to expensive healthcare facilities or long stays in these facilities. In addition, smartphone-based healthcare systems offer a cost-effective alternative for long-term health monitoring and early detection. They allow patients as well as healthcare professionals to remotely monitor and evaluate their patients without interfering with their daily activities. For example, lung cancer has been studied for decades due to its high mortality. Traditional methods including bronchoscopy and needle biopsy are notoriously invasive and expensive, exposing patients to increased risk and cost. Several noninvasive lung cancer biomarkers, such as exhaled breath condensates (EBCs), have been discovered for diagnosis and screening. However, their detection still relies on professional instruments that are limited to trained personnel or laboratories. This severely hinders population screening for lung cancer diagnosis, as well as all other cancers. Advanced smartphones integrated with powerful apps can provide easy operation and real-time monitoring for healthcare services. Zhang et al. developed an immunosensor to measure proteins in EBCs. They reported a miniaturized platform that provides online detection of carcinoembryonic antigen (CEA) in EBCs [134].

In addition, as already mentioned, POC sensors based on smartphones have attracted great interest due to their ability to function as fast and on-site testing tools without the need for complex instrumentation and a healthcare professional, as well as saving labor, reducing cost, and processing time. Therefore, especially in recent years, researchers have started to integrate analytical tools for data processing and communication based on colorimetric and electrochemical detection techniques into smartphone systems by taking advantage of the advanced features of smartphones. Compared to laboratory-based diagnostics, optical or electrochemical biosensors with smartphone add-ons are more suitable for POC use for on-site disease diagnosis and personal health monitoring. Colorimetric sensing has been made possible by smartphone cameras, while other technical capabilities of smartphones in the field of biosensing include data display and processing to meet different needs depending on the sensing method. These platforms are used for healthcare applications such as surgical diagnosis and self-diagnosis. In addition, the monitoring of various biomarkers, as discussed in this review, is becoming accessible to patients without the need for a healthcare professional. Smartphones have gained the ability to provide biosensing technologies such as direct measurement systems, results display, and telemedicine. Researchers are providing these technologies through smartphone apps and tools. Advanced smartphones integrated with powerful apps can provide easy operation and real-time monitoring for healthcare services.

*Signal detection and data analysis using a smartphone*. The high-resolution optical camera built into the smartphone is used as a visual imaging detector for analysis and detection applications by capturing the output signal, usually fluorescence, colorimetric, etc., that is triggered by the target analyte. Images can be obtained directly by photography or captured in a video recording at fixed time intervals. Then, depending on the analyte to be detected, they are processed with the help of a self-developed application or applications such as ImageJ and Adobe Photoshop, which can be installed for free on smartphones. As mentioned above, smartphone-based microfluidic devices are not only optical or electrochemical detectors but also provide image analysis and processing through applications developed according to the areas to be diagnosed. For example, smartphone-supported microfluidic sensors are used for automatic image and result processing using different applications, such as Color Grab, Hunt Color, and RGB color detector. Recent research has focused on developing applications that automatically process signals from microfluidic sensors and output the results. These applications, which we can download for free on our phones and use to get results, can be negatively affected by many factors such as the shooting angle, the light of the environment in which the images are recorded, or the source of excitation, which can lead to inaccurate results. For this reason, Song et al. developed a special Android app by combining isothermal amplification (BART-LAMP) technology with smartphone detection technology, eliminating the need for excitation sources and optical filters required in fluorescence detection [135]. To demonstrate the utility of this application, they were able to quantitatively detect the Zika virus in urine and saliva and the HIV virus in blood within 45 min. On the other hand, Hussain et al. produced a POC device integrated into a smartphone for optical, in situ, and rapid monitoring of clinically important serum albumin (SA) proteins based on color change with certain chemicals [136]. It is thought that these studies will shed light on cancer biomarker detection studies.

*Smartphone as a results display tool.* The research has often only functioned as a signal display, similar to the screen interface of the device for electrochemical detection, but the smartphone can also be used as a controller and to manipulate experimental devices. This has been achieved through the use of the smartphone as an interface device for analytical devices via Wi-Fi, Bluetooth, cloud, or MicroUSB. The use of smartphone hardware and software integrated with miniaturized devices, achieved through the development of technology, represents one of the greatest prospects for POC testing today. Azahar Ali et al. integrated a microfluidic electrochemical biosensing device with a portable potentiostat and a smartphone via a USB-C port [137]. The signals obtained were processed by PStouch software, enabling the rapid detection of COVID-19 antibodies within seconds.

*Telemedicine.* Telemedicine is a technological development that promotes and guarantees remote medical care through electronic platforms that allow disease detection and patient monitoring. The data detected through a wearable biosensor or other biosensors is captured by a smartphone/laptop and the detected data is sent to a cloud to be stored, processed, and retrieved, and finally, the data can be displayed on another device with dedicated applications. As it is applied to smartphones, it is becoming an important subject of study, enabling the transfer of data from patient to doctor or from doctor to patient in a portable, easy-to-use way. The field of telemedicine is rapidly advancing, especially in the sphere of infectious diseases, in countries with inadequate health care, and filling the gaps in access to care for technologically deprived populations. Considering COVID-19, which has significantly affected the health of the world population in recent years, the existence of an application such as telemedicine was of great importance. In response, Torrente-Rodriguez et al. presented a multiplex, portable, and wireless electrochemical platform [138]. In line with this study, a device was developed to detect viral antigen nucleocapsid protein, IgM, and IgG antibodies as well as the inflammatory biomarker C-reactive protein using blood and saliva samples, and the results obtained could be easily transmitted to the patient and then to healthcare providers.

Despite all this promising work, studies on smartphone-based microfluidic devices for cancer biomarker detection are limited and still in the early stages of development.

## 7. Challenges and Future Prospects

In light of the above-discussed studies, it is predicted that the burden in the field of health can be reduced when smart devices become more integrated into our lives. μPADs, which are recyclable, portable, inexpensive, on-site, applicable in resource-limited places, and user-friendly, are expected to reduce mortality rates by enabling early diagnosis of cancer.

Currently, although many studies based on cancer biomarkers have been reported, there are no paper-based microfluidic kits widely used in practice. Further research on the advantages and disadvantages of μPADs and their design as commercial kits is needed to understand the limitations and find solutions. Large-scale studies with real patient samples should be conducted to improve the reproducibility and response times of the tests. In addition, the interpretation of the results should be more in line with the requirements of on-site diagnostic tests, and device designs that are more understandable and simpler for the end user should be preferred.

## 8. Conclusions

μPADs have the potential to change cancer diagnosis by providing a low-cost, portable, and easy-to-use alternative to traditional diagnostic methods. These POC devices work by using paper as the substrate for microfluidic channels, which can be used to isolate and analyze samples of body fluids such as blood or urine. Analyzing the biomarkers present in these samples allows the practical detection of either presence or absence of cancer. On the other hand, μPADs could be produced at low costs, making them accessible to a wider range of patients.

Traditional diagnostic methods, on the other hand, often require expensive equipment as well as a trained person, which can be an obstacle, especially for patients in resource-limited settings.

Another advantage of these systems is their portability in comparison with the traditional methods which require patients to travel to a well-equipped laboratory or hospital. Moreover, μPADs can be easily transported and administered in a variety of settings, including at home or in a doctor’s office.

In conclusion, μPADs have the potential to greatly improve cancer diagnosis by providing a low-cost, portable, and easy-to-use alternative to traditional methods. As research in this field continues to advance, it is likely that these tests will become increasingly widespread and will play a crucial role in the early detection and treatment of cancer.

## Data Availability

Not applicable.
